# High-quality genome assembly and comparative genomic profiling of yellowhorn (*Xanthoceras sorbifolia*) revealed environmental adaptation footprints and seed oil contents variations

**DOI:** 10.3389/fpls.2023.1147946

**Published:** 2023-03-21

**Authors:** Juan Wang, Haifei Hu, Xizhen Liang, Muhammad Tahir ul Qamar, Yunxiang Zhang, Jianguo Zhao, Hongqian Ren, Xingrong Yan, Baopeng Ding, Jinping Guo

**Affiliations:** ^1^College of Forestry, Shanxi Agricultural University, Taigu, Shanxi, China; ^2^Shanxi Key Laboratory of Functional Oil Tree Cultivation and Research, Shanxi Agricultural University, Taigu, Shanxi, China; ^3^Rice Research Institute, Guangdong Key Laboratory of New Technology in Rice Breeding, Guangzhou, China; ^4^Guangdong Rice Engineering Laboratory, Guangdong Academy of Agricultural Sciences, Guangzhou, China; ^5^Integrative Omics and Molecular Modeling Laboratory, Department of Bioinformatics and Biotechnology, Government College University Faisalabad (GCUF), Faisalabad, Pakistan; ^6^Engineering Research Center of Coalbased Ecological Carbon Sequestration Technology of the Ministry of Education, Datong University, Taigu, Shanxi, China

**Keywords:** yellowhorn, pan-genomics, genomic profiling, adaptation, oil contents

## Abstract

Yellowhorn (*Xanthoceras sorbifolia*) is a species of deciduous tree that is native to Northern and Central China, including Loess Plateau. The yellowhorn tree is a hardy plant, tolerating a wide range of growing conditions, and is often grown for ornamental purposes in parks, gardens, and other landscaped areas. The seeds of yellowhorn are edible and contain rich oil and fatty acid contents, making it an ideal plant for oil production. However, the mechanism of its ability to adapt to extreme environments and the genetic basis of oil synthesis remains to be elucidated. In this study, we reported a high-quality and near gap-less yellowhorn genome assembly, containing the highest genome continuity with a contig N50 of 32.5 Mb. Comparative genomics analysis showed that 1,237 and 231 gene families under expansion and the yellowhorn-specific gene family NB-ARC were enriched in photosynthesis and root cap development, which may contribute to the environmental adaption and abiotic stress resistance of yellowhorn. A 3-ketoacyl-CoA thiolase (*KAT*) gene (*Xso_LG02_00600*) was identified under positive selection, which may be associated with variations of seed oil content among different yellowhorn cultivars. This study provided insights into environmental adaptation and seed oil content variations of yellowhorn to accelerate its genetic improvement.

## Introduction

Yellowhorn (*Xanthoceras sorbifolia*), belonging to the *Xanthoceras* genus (*Sapindaceae* family), is a unique woody tree plant species widely growing in Northern and Central China (Bi et al., 2019; Liu et al., 2021a). Yellowhorn shows strong abiotic stress resistance ability and can grow under extreme environmental conditions, including extreme temperature, drought conditions, saline, and alkaline land (Ruan et al., 2017). Furthermore, yellowhorn is easy to reproduce, sowing, root cutting, and grafting and is now considered a promising afforestation species for many arid areas. This oil-rich tree produces capsular fruits from hermaphrodites, with about 60% of its seed kernel containing edible seed oil for the human diet and around 4% nervonic acid essential for nerve and brain development with high medicinal and ornamental value (Liang et al., 2019; Liang et al., 2022). However, yellowhorn also contains moderate erucic acid (about 9% of the total fatty acid) that can damage the heart at high doses (Liu et al., 2021b). Therefore, to make yellowhorn a more desirable species for oil production, it is essential to underly the genetic basis of its oil synthesis pathway and design and cultivate new species with a lower level of erucic acid and a higher level of nervonic acid.

The 3-ketoacyl-CoA thiolase (KAT) is a member of thiolase and can catalyze the final step of fatty acid β-oxidation and the claisen condensation reaction between two Acetyl-CoAs and lead to carbon chain elongation, which is a key step in the fatty acid biosynthetic pathways (Footitt et al., 2007). So far, KAT has been reported to play an important role in producing various energy-storage molecules, such as fatty acids and affecting seed oil content and synthesis in *Arabidopsis thaliana* (Germain et al., 2001) and *Jatropha curcas* (Gomes et al., 2010). However, the mechanism of KAT regulation in yellowhorn and how it underly the fatty acid synthesis remains to be elucidated.

The rapid development of sequencing technologies has facilitated the development of yellowhorn genomes, with two good-quality yellowhorn genome assemblies being published recently (Liang et al., 2019; Liang et al., 2022). These published yellowhorn genomes were sampled and collected from a valley terrain environment with mountains and rivers in Shandong Province. However, yellowhorn also grew and adapted to the loess plateau with a more extreme climate. Therefore, in this study, using long-read sequencing, we sequenced and assembled a gapless *Xanthoceras sorbifolia* genome of the superior line “G11” (Data named *XsoG11*), which was collected from the loess plateau located in Shanxi province. By performing the comparative genomic analysis among representative angiosperms, we revealed that gene families with functions of photosynthesis and root cap development were expanded and existed in yellowhorn, which may associate with adaptation to extreme environmental conditions. With the availability of high-quality yellowhorn reference genomes, we performed the pangenome-wide analysis among three yellowhorn genomes and identified gene content variations that may associate with environment adaptation and oil content variations of different yellowhorn cultivars. All the above results will provide new insights into genetic diversity study of yellowhorn and helps in its genetic improvement.

## Materials and methods

### Plant materials

*Xanthoceras sorbifolia* superior cultivar “XsoG11” (*Xanthoceras sorbifolia* superior G11) is a strain with highly comprehensive evaluation selected by *Xanthoceras sorbifolia* research group of Shanxi Agricultural University collected from Lvliang Mountain (Shanxi province; 111°47′17″East, 37°15′57″North) (**Supplementary Figure 1**), located in semi-arid area, which is extremely cold in winter. The DNA sequencing libraries of PacBio HiFi long reads, Illumina short reads, and Hi-C reads were prepared according to the standard Illumina and PacBio library construction protocol for the generation of genome assembly “XsoG11” (Liang et al., 2021).

### Genome assembly

The clean PacBio HiFi reads were assembled using Hifiasm (v.0.15) (Cheng et al., 2021) with default parameters. Then, the original assembly result is polled using pilon (v1.23) (Walker et al., 2014) to get the final genome assembly result. Chromosome-length scaffolds were generated by aligning the raw HiC-reads to the draft assembly using Juicer (v.1.6) (Durand et al., 2016) with the resulting alignments processing by the 3D-DNA pipeline (v.19) (Dudchenko et al., 2017) to generate the candidate chromosome-length assemblies. This candidate assembly was reviewed and curated using Juicebox Assembly Tools (v.1.11.08) (Robinson et al., 2018). BUSCO V3 (Simao et al., 2015) with eukaryota_odb9 was used to assess the completeness of the assembly.

### Repeat sequence annotation

For the repeat sequence annotation, trf (v4.09) (Benson, 1999) was used to predict tandem repeats; Microsatellite sequence uses misa Pl program prediction; LTR First use LTR separately_Finder and LTR_Harvest Identify, then use LTR_ Retriever (v2.7) (Ou and Jiang, 2018) integrates the results of the above two software to obtain the final LTR identification results; LINE, SINE, and DNA transposons were identified by RepeatMasker (v4.0.9) (Tarailo-Graovac and Chen, 2009). The two methods are combined to identify the repeat contents in our genome, homology-based and *de novo* prediction. Homology-based analysis: We identified the known TEs within the XsoG11 genome using RepeatMasker (v4.0.9) (Tarailo-Graovac and Chen, 2009) with the Repbase TE library. *De novo* prediction: We constructed a *de novo* repeat library of the XsoG11 genome using RepeatModeler, which can automatically execute two core *de novo* repeat-finding programs, namely, RECON (v1.08) (Bao and Eddy, 2002) and RepeatScout (v1.0.5) (Benson, 1999), to comprehensively conduct, refine and classify consensus models of putative interspersed repeats for the XsoG11 genome. Furthermore, we performed a *de novo* search for long terminal repeat (LTR) retrotransposons against the XsoG11 genome sequences using LTR_Finder (v1.0.7) (Xu and Wang, 2007), LTR_harvest (v1.5.11) and LTR_retriever (v2.7) (Ou and Jiang, 2018). We also identified tandem repeats using the Tandem Repeat Finder (TRF) package and the SimpleSequence Repeats (SSR) using misa (v1.0) (Beier et al., 2017). Finally, we merged the library files of the two methods to identify and determine the repeat contents.

### Gene annotation

We predicted protein-coding genes of the XsoG11 genome using three methods: ab initio gene prediction, homology-based gene prediction, and RNA-Seq-guided gene prediction. Before gene prediction, the assembled XsoG11 was hard and soft masked using RepeatMasker (v4.0.9) (Tarailo-Graovac and Chen, 2009). We adopted Augustus (v3.3.3) (Stanke et al., 2008) to perform *ab initio* gene prediction. Models used for each gene predictor were trained from a set of high-quality proteins generated from the RNA-Seq dataset. We used maker (v2.31.10) (Holt and Yandell, 2011) to conduct homology-based gene prediction. First, the protein sequences and transcripts sequences were aligned to our genome assembly and predicted coding gene using maker with the default parameters. To carry out RNA-Seq-guided gene prediction, we first aligned clean RNA-Seq reads to the genome using hisat2 (v2.0.0) (Kim et al., 2015), and the gene structure was formed using Trinity (v2.3.2) (Grabherr et al., 2011), Transdecoder (v2.01) (Haas et al., 2017) and maker (v2.31.10) (Holt and Yandell, 2011). Finally, EVidenceModeler (v1.1.1) (Haas et al., 2008) was used to integrate the prediction results of the three methods to predict gene models. Functional annotation was performed by comparing proteins with various functional databases including NR, swiss pro, KOG and TrEMBL, using BLASTP (e-value < 1e-5) (Camacho et al., 2009).

### Comparative genomics analysis

Using the assembled yellowhorn genome (XsoG11) and nine other related angiosperm genomes, we performed a comparative genome analysis using OrthoFinder (v 2.4.0) (Emms and Kelly, 2019) to identify the orthologous gene families in the yellowhorn genome. The analysis process of the OrthoFinder was indicated as follows: 1) Use the diamond to input all protein sequences for all-*vs*-all comparison and detect homologous gene pairs (Evaluate < 1e-5 and the minimum coverage is > 40%). 2) Input the list of homologous gene pairs into MCL program for family clustering. A maximum likelihood phylogenetic tree of ten species was constructed based on shared single-copy genes using Mega V5 (Tamura et al., 2011). Expanded and contracted gene families were detected using CAFÉ (v4.2.1) (De Bie et al., 2006). The expanded gene families were functionally annotated on Pfam v32.0 (Mistry et al., 2021) and Swiss-Prot (UniProt, 2021) databases. The functional enrichment of each gene family was determined using a Fisher’s exact test (false discovery rate < 0.05).

### Pan-genomics analysis

The genome sequences and protein sequences of two published yellowhorn (WF18 and Xsv2) were downloaded from Liang and Liu study (Liang et al., 2019, Liu et al., 2021a). Orthologous genes among the yellowhorn genomes were identified by Orthovenn2 (Xu et al., 2019), a web tool used to identify orthologous and paralogous genes, with a pairwise sequence similarity cut-off of 10- 5 and inflation of 1.5 to define orthologous cluster structure. KaKs_Calculator 2.0 (Wang et al., 2010) was used to calculate orthologous gene clusters’ non-synonymous/synonymous substitution ratio. Orthologous clusters and gene pairs under positive selection (Ka/ks > 1) were analyzed by UniProt search and TopGO (Alexa et al., 2006) using Fisher’s exact tests for functional annotation and enrichment analysis. Furthermore, the two published genomes were compared to our genome assembly using the mumandco_ V3 program (parameter default) (O’donnell and Fischer, 2020).

## Results

### Genome assembly and annotation

**Figure 1:**
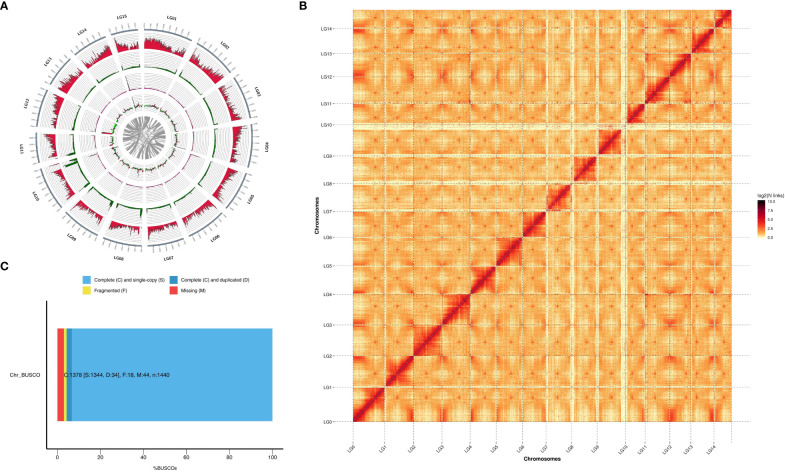
The characteristics of yellowhorn XsoG11 genome assembly. **(A)** The yellowhorn genome feature. From outer-most track to innermost track: gene density, transposable element density, repeat sequence density, GC content, and Intra-genome collinear blocks. **(B)** Buscos of complete genome. **(C)** Contact map of Hi-C links among 14 pseudochromosomes.

In this study, we used the long read sequencing to *de novo* assemble a near gapless genome assembly of the Shanxi yellowhorn cultivar XsoG11 (**Figure 1A**; **Table 1**). We generated approximately 30-fold coverage of PacBio CCS (HiFi) reads and assembled the CCS (HiFi) reads using Hifiasm: a haplotype-resolved assembler for accurate HiFi reads (Cheng et al., 2021). The assembly length of the XsoG11 genome is 489.18 Mb with a contig N50 of 32.5 Mb, showing the highest contiguity than the previously published yellowhorn genomes (**Table 2**). The contigs were further polished using pilon, then ordered, oriented and anchored to chromosomes using *in-situ* Hi-C sequencing. We found that around 96.2% (470.79 Mb) of sequences are anchored to the chromosome and seven chromosomes in our genome do not contain any gaps (**Table 1**; **Figure 1B**). XsoG11 assembly has 95.7% complete BUSCOs (**Table 2**; **Figure 1C**), comparable to the previously published Xsv2 (Liu et al., 2021b) and WF18 yellowhorn genomes (Liang et al., 2019). In addition, we identified approximately 68.71% repeat sequences in the assembled genome, in which the long terminal repeat (LTR) retrotransposon element represents the most abundant transposable elements (TEs) class, accounting for 35.8% TEs (**Table 3**). Using RNA-seq transcript mapping combined with ab initio prediction and homologous protein searches, we predicted 35,039 protein-coding genes with an average gene length of 2,662 bp in the XsoG11 genome (**Supplementary Table 1**), in which 27,082 (85%) genes have functional annotation from at least one functional protein database, including nr (84%), TrEMBL (84%), KOG (41%), Swiss-Prot (57%), Pfam (69%), Gene Ontology (GO) (42%), and KEGG (23%) (**Supplementary Table 2**). We also identified 1,250 tRNAs, 770 small nucleolar RNAs and 4,691 small nuclear RNAs (**Supplementary Table 3**).

**Table 1:** Genome Statistic of *Xanthoceras sorbifolia* superior G11.

Chr	Chr_size(bp)	Contig_number	Contig_size(bp)	GC_content(%)
LG01	39494243	1	39494243	34.9
LG02	35873774	1	35873774	35.5
LG03	35257883	4	35257583	35.05
LG04	34944601	2	34944501	35.14
LG05	32593928	4	32593628	34.99
LG06	32495627	1	32495627	35.18
LG07	29437501	1	29437501	35.28
LG08	31632995	1	31632995	35.22
LG09	31780288	3	31780088	35.34
LG10	35671781	2	35671681	35.4
LG11	24152185	3	24151985	35.44
LG12	30754806	7	30754206	36.83
LG13	26198562	1	26198562	35.16
LG14	28601909	1	28601909	35.09
LG15	21899742	2	21899642	35.21
ChrAll	470789825	34	470787925	35.31

**Table 2:** Statistic of different yellowhorn genome assemblies.

TypeParameter	WF18v1	Xsv2	ZS4	WF18	XsoG11
Assembly Genome size(Mb)	490.44	470	504.2	440	489.18
Chromosome-scale scaffolds(Mp)	490.24 (99.96%)	446.2 (94.9%)	489.29 (97.04%)	420 (95.4%)	470.79 (96.24%)
Total num. of scaffolds	22	988	297	267	417
Total num. of chromosomes	15	15	15	15	15
ScaffoldN50(Mb)	34	30.8	32.17	29.4	31.6
Total num. of Contigs	2,428	3,302	3,035	2,002	417
Contig N50(Mb)	0.42	0.42	1.04	0.64	31.6
Complete BUSCOs	98.70%	97.50%	98.70%	84.60%	95.70%
GC content of the genome(%)	34.71	34.94	36.95	32.75	35.70%
Protein-coding genes	29,888	22,049	24,672	21,059	35,039
Reference	(Liang et al., 2022)	(Liu et al., 2021a)	(Bi et al., 2019)	(Liang et al., 2019)	This study

**Table 3:** Repeat elements of the XsoG11 yellowhorn genome.

repeat_type	total_size	Percentage of genome (%)
DNA/CMC-EnSpm	1456919	0.30%
DNA/hAT-Ac	5043893	1.03%
DNA/hAT-Tag1	1039259	0.21%
DNA/hAT-Tip100	1516665	0.31%
DNA/MuLE-MuDR	4508774	0.92%
DNA/PIF-Harbinger	887491	0.18%
DNA/TcMar-Pogo	209047	0.04%
LINE/L1	19514034	3.99%
LINE/RTE-X	67436	0.01%
Low_complexity	1285729	0.26%
LTR	1516161	0.31%
LTR/Caulimovirus	1471510	0.30%
LTR/Copia	46538428	9.51%
LTR/Gypsy	62413913	12.76%
Simple_repeat	8520634	1.74%
Unknown	180122927	36.82%
Total	336112820	68.71%

### Yellowhorn phylogenetics and gene family expansion analyses

**Figure 2:**
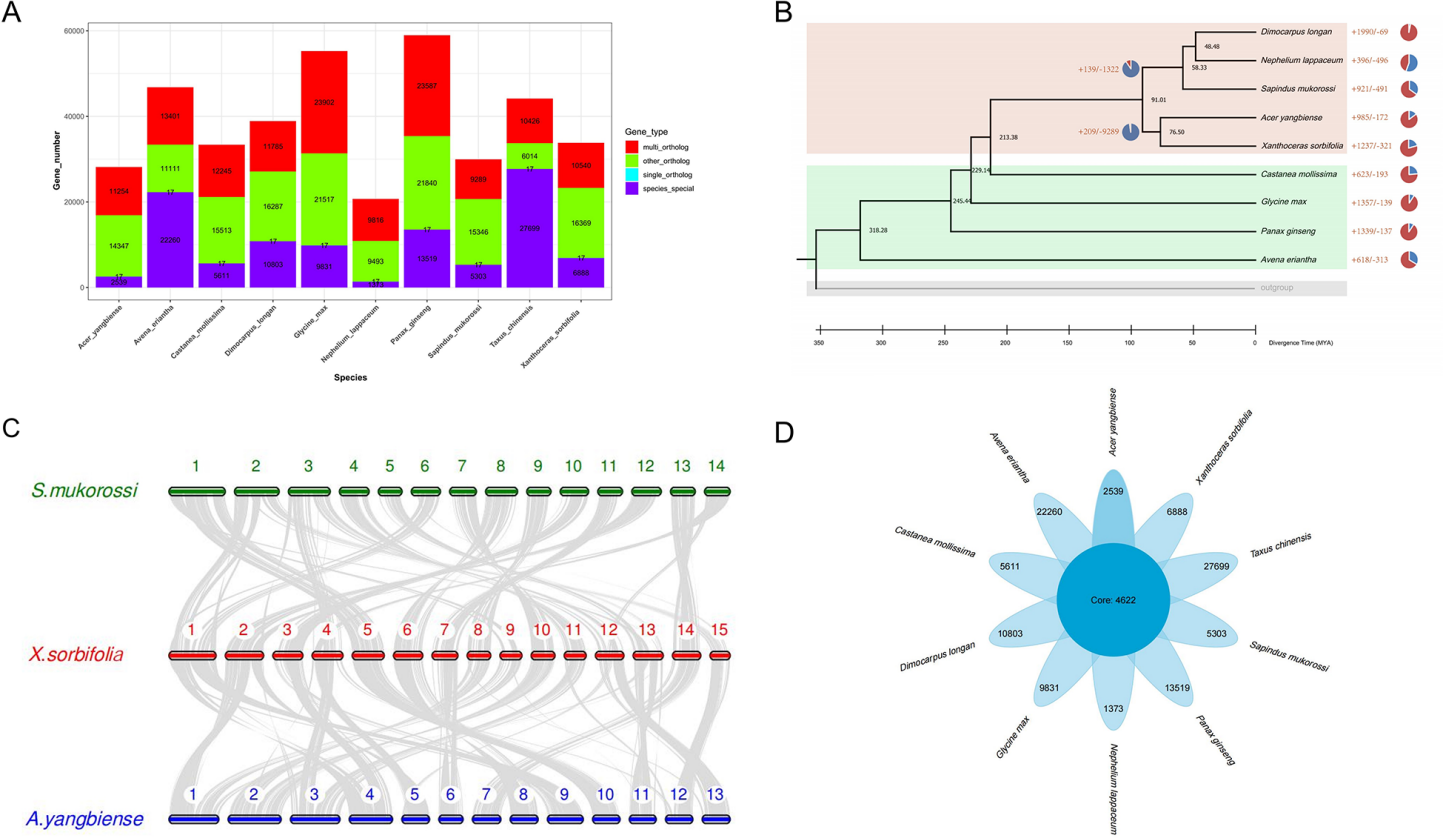
Comparative analysis yellowhorn and nine other representative angiosperms. **(A)** Gene family clusters. Multi_ortholog: This family exists in all species, and the number of members in at least one species is greater than or equal to two; single_ortholog: a single copy orthologous gene family, which exists in all species and has 1 member in all species; other_ortholog: The family exists in at least two species and does not exist in at least one species; species_special: the unique gene family of each species. **(B)** Maximum-likelihood phylogeny of yellowhorn and nine other representative angiosperms. **(C)** Collinear analysis by comparing the yellowhorn (*X*.sorbifolia, XsoG11) genome with *Acer yangbiense* (*A. yangbiense*) and *Sapindus mukorossi* genomes (*S. mukorossi*). **(D)** The comparative genomic analysis shows the number of the core and species specific gene families.

The change in gene family size plays an important role in the evolution of angiosperms’ environmental adaptation and trait formation during evolution (Van de Peer et al., 2009). To further dissect the genetic basis of high seed oil content and the ability to adapt to extreme environments of yellowhorn, we performed the comparative genomics and gene family expansion analysis in yellowhorn and eight other representative angiosperms and the outgroup species (*Taxus chinensis*). We first determined the phylogeny position of yellowhorn by constructing a phylogenetic tree using 17 single-copy orthologous genes conserved in 10 representative angiosperms (**Figure 2A**; **Supplementary Table 4**). Our result inferred that *Acer yangbiense* was the most recent common ancestor of yellowhorn, which diverged around 76.5 million years ago (**Figure 2B**).

We further performed the collinear analysis by comparing the yellowhorn (XsoG11) genome with *Acer yangbiense* and *Sapindus mukorossi* genomes (**Figure 2C**). Although these species belong to the *Sapindaceae* family, we identified significant structural variations in yellowhorn and *Acer yangbiense* and *Sapindus mukorossi*, suggesting that significant chromosomal differentiation occurred since they derived from the last common ancestor. A total of 4,622 gene families were shared by ten studied species (**Figure 2D**), with yellowhorn having 6,888 species-specific gene families. GO enrichment analysis indicates that these species-specific gene families are significantly enriched in functions associated with photosynthesis (**Supplementary Table 5**). Additionally, the gene family size analysis showed that 1,237 and 231 gene families were found to be expanding and contracting (**Supplementary Table 6**). Enrichment analysis showed that the expanding gene families were enriched for functions associated with disease resistance (Pfam: NB-ARC domain) (**Supplementary Table 7**) and photosynthesis (GO: “photosynthetic electron transport in photosynthesis”, “photosynthetic electron transport chain”, “photosynthesis, light reaction” and “photosynthesis”; KEGG: ko00195: Photosynthesis) (**Supplementary Tables 8, 9**). These results may suggest that gene family expansion was associated with photosynthesis and biotic stress resistance in yellowhorn.

### Pan-genome analysis of yellowhorn genomes

**Figure 3:**
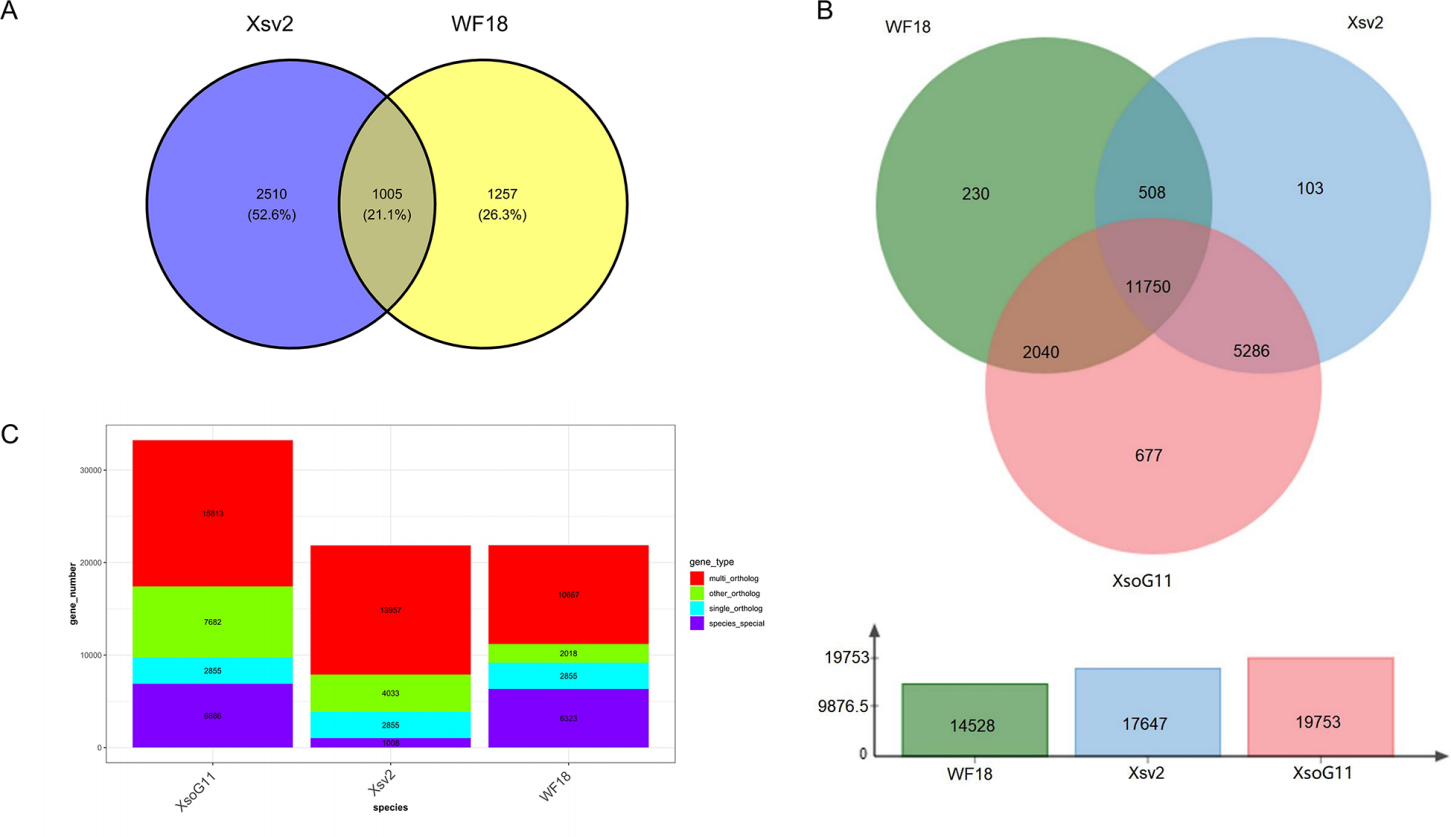
Pan-genome analysis of yellowhorn genomes. **(A)** The Venn diagram shows the number of SVs that are uniquely and commonly absent in the Xsv2 and WF18 genome, by comparing with the XsoG11 genome. **(B)** Pangenome-wide analysis of the core and dispensable gene families among three different yellowhorn genomes. **(C)** Gene family clustering,.

**Figure 4:**
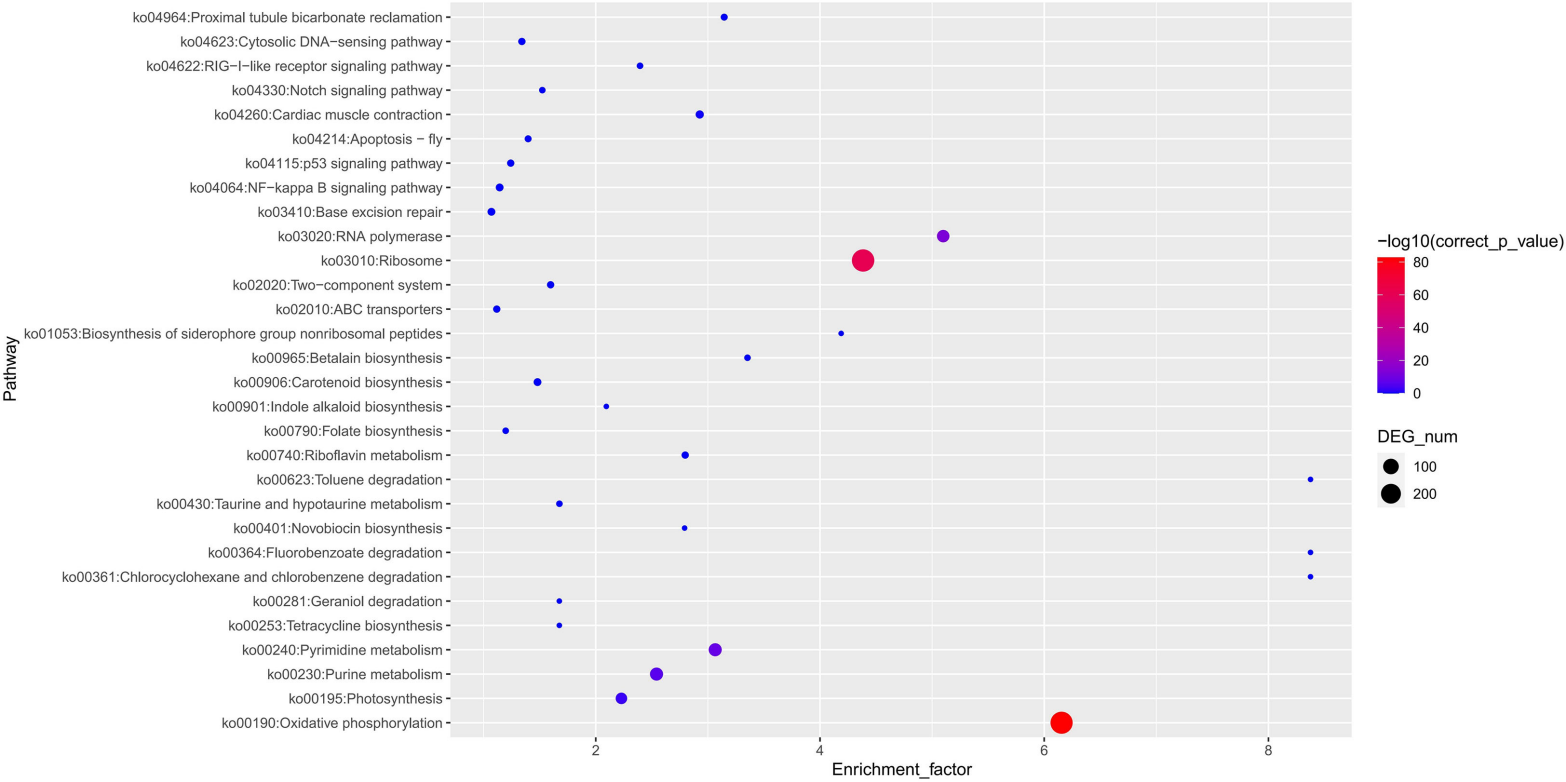
XsoG11 special gene enrichment in KEGG.

High-quality genome assemblies enable the accurate discovery of structural variations and genetic variations among genomes. Using the high-quality genome assembly (XsoG11) as the reference genome, we further discovered abundant structural variations (SVs), including inversions, translocations, insertions and deletions between the XsoG11 genome and the other two published yellowhorn genomes (Xsv2 and WF18) (**Figure 3A**; **Supplementary Tables 10, 11**). We identified 3,515 and 2,262 sequences uniquely present in XsoG11 by comparing this assembly with Xsv2 and WF18 genomes, respectively, in which 1,005 sequences are present in the XsoG11 genome but missing in both Xsv2 and WF18 genomes (**Figure 3B**). In addition, we further performed the pan-genome wide analysis of gene families (Zia et al., 2022). The pangenome-wide gene family clustering analysis revealed that these three genomes shared 11,750 core orthologous clusters, whereas at least one genome (but less than three) shared 8,844 dispensable orthologous clusters, with 677 XsoG11-specific dispensable orthologous clusters (**Figure 3C**). The evolutionary analysis of the three yellowhorn showed that they are clustered together (**Supplementary Figure 2**) and the results of collinearity is consistent (**Supplementary Figure 3**). Functional annotation of genes located in these 1,005 sequences found oxidative phosphorylation (ko00190, *P* < 2.19E-83), ribosome (ko03010, *P* < 1.06E-61), RNA polymerase genes (ko03020, *P* < 1.44E-13) were the first three most significantly enriched in KEGG pathyway. In addition, photosynthetic pathway genes were also significantly enriched (ko00195, *P* < 0.003) (**Figure 4**). Gene ontology (GO) enrichment analysis shows that genes with essential biological functions, including RNA-DNA hybrid ribonuclease activity, DNA recombination, oxidation-reduction process and DNA integration, were enriched in core orthologous clusters (**Supplementary Table 12**). By contrast, genes with functions potentially associated with fatty acid synthesis and abiotic stress responses, such as photosynthesis and root cap development, are enriched in XsoG11-specific dispensable orthologous clusters (**Supplementary Table 13**). We further examined the non-synonymous/synonymous substitution ratio (Ka/Ks) of homologous gene pairs of XsoG11 and two other published yellowhorn genomes (Xsv2 and WF18). The result showed that a total of 364 genes in the XsoG11 are under positive selection (Ka/Ks > 1) (**Supplementary Table 14**), including a gene (*Xso_LG02_00600*) encoding 3-ketoacyl-CoA thiolase associated with the formation of fatty acid (ko01040: Biosynthesis of unsaturated fatty acids; ko00592, alpha-Linolenic acid metabolism).

## Discussion

A high-quality yellowhorn genome assembly is key to underlying the genetic basis of its ability to adapt to extreme environments and produce high oil seed content. In this study, using Circular consensus sequencing (CCS) long-read sequencing, we present a high-quality chromosome-scale genome assembly of Shanxi’s yellowhorn cultivar (XsoG11). Compared with the previously published yellowhorn Shandong’s cultivar “Shanyou 1” genome (WF18) (Liang et al., 2021), we assembled a higher contiguity (Contig N50: XsoG11: 31.6 Mb *vs* WF18v1: 0.42 Mb) and a near-gapless yellowhorn genome, with seven out of 15 chromosomes having no gaps. This additional high-quality genome can provide novel genomic resources for future yellowhorn improvement.

Photosynthesis is an important physiological process that converts light energy into chemical energy, affecting plant growth and development, respiration and transpiration (Goudriaan et al., 1985). Recent studies suggest that genes involved in the photosynthesis pathway are essential for environmental adaptation to different light regimes and coping with climate change by regulating the circadian clock and light perception (Kreps and Kay, 1997; Quint et al., 2016). For example, a pan-genome study of mung beans indicates that the presence/absence variation (PAV) of genes regulating the photosynthesis pathway enables mung beans to adapt to different environments (Liu et al., 2022). Gene expansion through tandem duplication is important for stress response (Hanada et al., 2008). Photosynthesis-related genes, early light-induced proteins (ELIPs), were found to be expanded in plants showing drought resistance (Liu et al., 2020). Combined with the public genome assemblies of representative angiosperms and yellowhorn, this high-quality assembly enabled us to identify gene family differences and the expansion/contraction of yellowhorn during speciation and divergence and among yellowhorn cultivars growing in a different climate region. Compared with other angiosperms and yellowhorn cultivars collected in Shandong Province, the yellowhorn assembled in this study grows in the Loess Plateau of the middle of Shanxi Province with poor and dry soil (Yao et al., 2013). Both GO enrichment analyses of yellowhorn’s expanding gene families and pangenome-wide XsoG11-specific dispensable gene families showed that gene families are significantly enriched in functions associated with photosynthesis. The enrichment of photosynthesis can lead to the accumulation of plant carbohydrates and other carbon sources, resulting in the generation of more energy to cope with the adverse stress of environmental factors in the Loess Plateau (Farrar and Williams, 1991). In addition, we also found that genes associated with root cap development were also enriched in the XsoG11-specific dispensable gene families that were missing in other yellowhorn cultivars. This may reflect that the yellowhorn cultivar growing in the drought Loess Plateau requires a more robust and extended root cap system (Du et al., 2011).

We also revealed that a gene (*Xso_LG02_00600*) encoding 3-ketoacyl-CoA thiolase (KAT) was under positive selection by comparing the value of Ka/Ks of homologous gene pairs of our yellowhorn cultivar with the Shandong’s yellowhorn cultivars. KAT is an important catalyst for the process of fatty acid beta-oxidation. In *Arabidopsis thaliana*, the *KAT* gene was demonstrated to be activated in the early germination and seedling stage and led to fatty acyl-CoAs accumulation and the form of triacylglycerol, facilitating lipid storage in the oil seed (Germain et al., 2001; Footitt et al., 2007). A similar finding was reported in *Ophiocordyceps sinensis* that KAT was regulated and participated in the fatty acid pathway and provided sufficient energy for organisms by catalyzing the tricarboxylic acid cycle and electronic respiratory chain (Wang et al., 2022). The positive selection of the *KAT* gene in our Shanxi’s yellowhorn cultivar may associate with its seed oil content variations among the comparative yellowhorn cultivars.

## Conclusion

In a nutshell, we assembled a high-quality and near gap-less yellowhorn genome collected from Loess Plateau, providing valuable genomic resources for future yellowhorn genetic improvement. The functional analysis shows that the gene family under expansion and the yellowhorn-specific gene family are enriched in functions associated with photosynthesis and root cap development, which may relate to the environmental adaption of yellowhorn. A *KAT* gene under positive selection was identified, reflecting variations of seed oil content among different yellowhorn cultivars. This study provide a foundation for further genetic improvement of yellowhorn.

## Data availability statement

All sequencing data and genome assembly generated in this study are available on SRA at the NCBI with the accession numbers PRJNA924504.

## Author contributions

JG and BD designed the experiment. JW and HH performed research and did the methodology, finish writing and editing. XL, YZ and JZ helped with the part of data and results. MTQ, HR and XY critically revised the manuscript. All authors contributed to the article and approved the submitted version.

## Supplementary material

Supplementary Figure 1 XsoG11 yellowhorn tree used in this study.

Supplementary Figure 2 Evolution tree of three yellowhorn (“WF18”, “XsoG11” and “Xsv2”).

Supplementary Figure 3 The collinearity of three yellowhorn (“WF18”,”XsoG11” and “Xsv2”).
