# Supplementary figures and images for: High-quality genome assembly and comparative genomic profiling of yellowhorn (Xanthoceras sorbifolia) revealed environmental adaptation footprints and seed oil contents variations
Source: Front Plant Sci. 2023 Mar 21;14:1147946. doi: 10.3389/fpls.2023.1147946 (PMC10070836; doi:10.3389/fpls.2023.1147946)

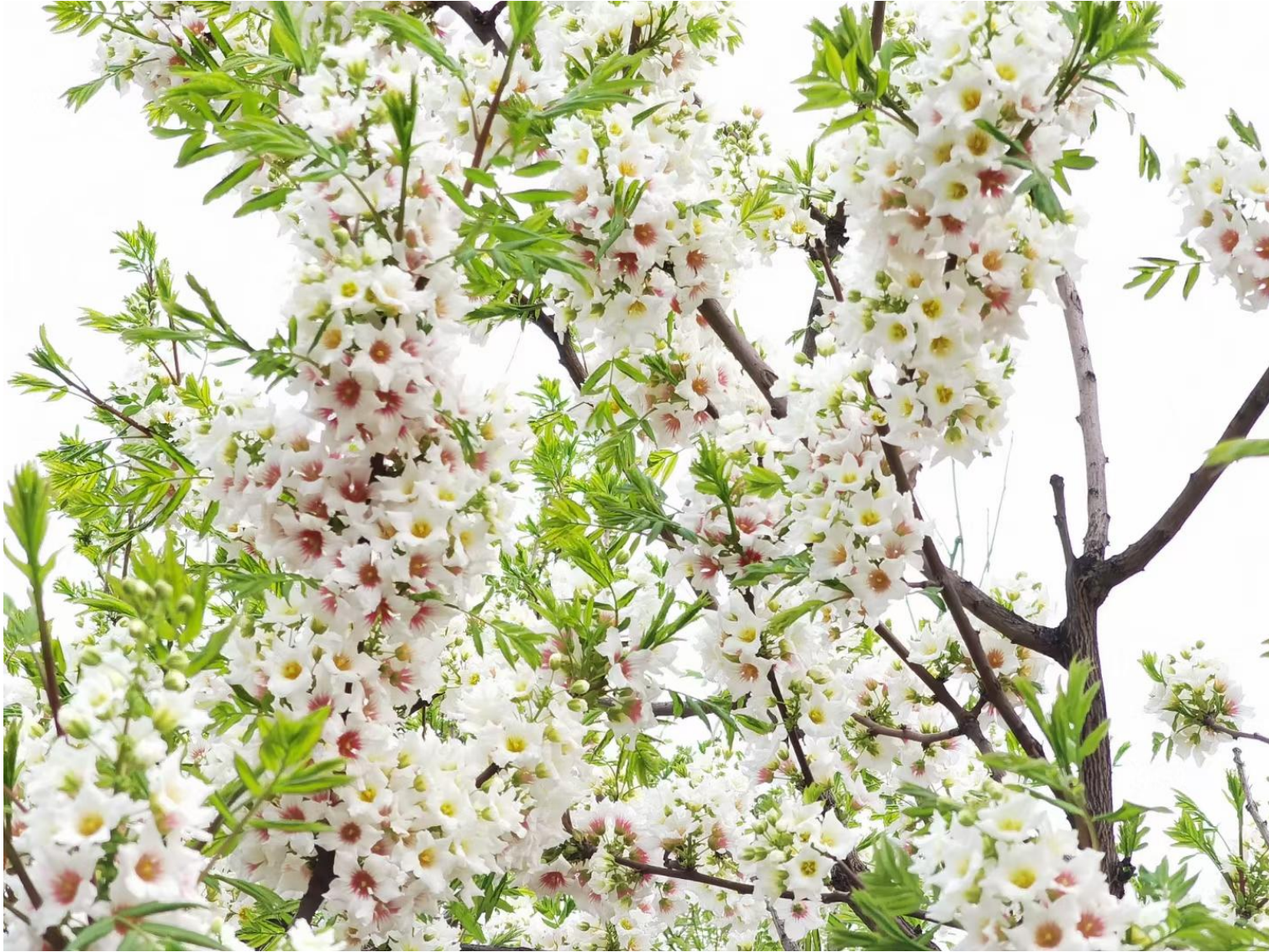

Supplement: Supplementary Figure 1 — XsoG11 yellowhorn tree used in this study. [file DataSheet_1.zip › Supplementary Figure 1.pdf]

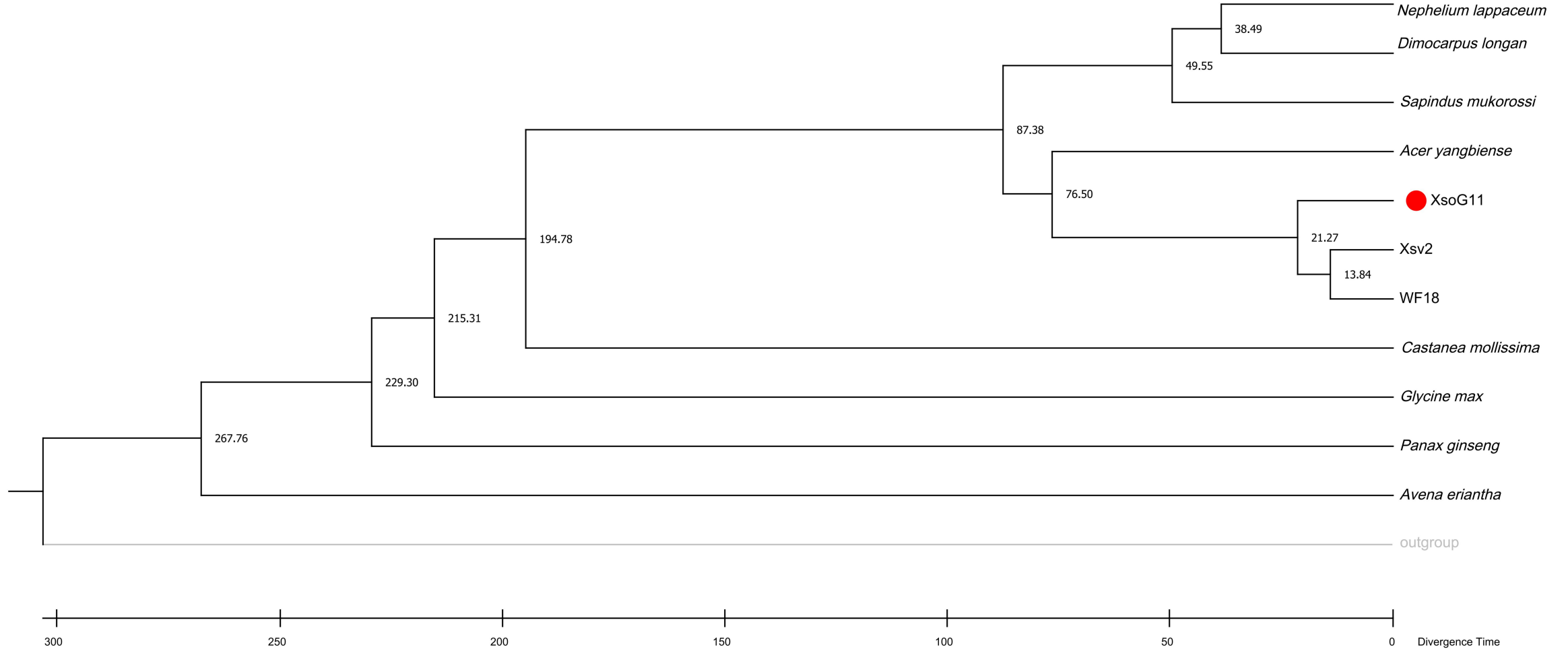

Supplement: Supplementary Figure 1 — XsoG11 yellowhorn tree used in this study. [file DataSheet_1.zip › Supplementary Figure 2.pdf]

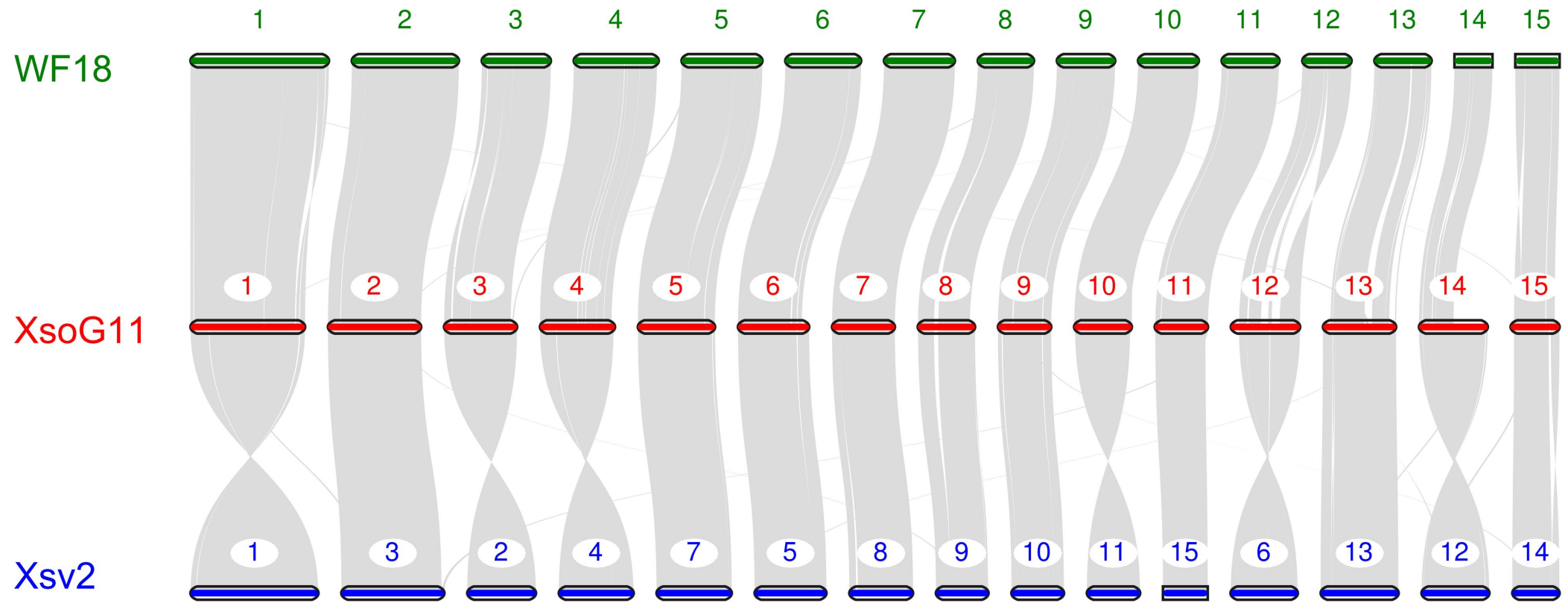

Supplement: Supplementary Figure 1 — XsoG11 yellowhorn tree used in this study. [file DataSheet_1.zip › Supplementary Figure 3.pdf]
